# NbThermo: a new thermostability database for nanobodies

**DOI:** 10.1093/database/baad021

**Published:** 2023-04-12

**Authors:** Mario S Valdés-Tresanco, Mario E Valdés-Tresanco, Esteban Molina-Abad, Ernesto Moreno

**Affiliations:** Faculty of Basic Sciences, University of Medellin, Cra. 87 No. 30-65, Medellin 050026, Colombia; Centre for Molecular Simulations and Department of Biological Sciences, University of Calgary, 2500 University Drive N.W, Calgary, AB T2N 1N4, Canada; Globant S.A., Av. El Poblado #5A 113, Medellin 050021, Colombia; Faculty of Basic Sciences, University of Medellin, Cra. 87 No. 30-65, Medellin 050026, Colombia

## Abstract

We present NbThermo—a first-in-class database that collects melting temperatures (*T*_m_), amino acid sequences and several other categories of useful data for hundreds of nanobodies (Nbs), compiled from an extensive literature search. This so-far unique database currently contains up-to-date, manually curated data for 564 Nbs. It represents a contribution to efforts aimed at developing new algorithms for reliable *T*_m_ prediction to assist Nb engineering for a wide range of applications of these unique biomolecules. Nbs from the two most common source organisms—llama and camel—show similar distributions of melting temperatures. A first exploratory research that takes advantage of this large data collection evidences that understanding the structural bases of Nb thermostability is a complex task, since there are no apparent differences in sequence patterns between the frameworks of Nbs with lower and higher melting temperatures, indicating that the highly variable loops play a relevant role in defining Nb thermostability.

**Database URL** https://valdes-tresanco-ms.github.io/NbThermo

## Introduction

Heavy-chain antibodies (HCAbs), commonly known as nanobodies (Nbs), have been gaining ground in the therapeutic and diagnostic areas since their discovery three decades ago. Common Nb applications include therapeutic uses in cancer and other diseases, *in vitro* and *in vivo* diagnosis, identification of molecular targets, drug delivery and as chaperone molecules in protein crystallography, among many others (1–4). Such versatility finds support in the unique structural characteristics of Nbs, such as the high thermal and reductive stabilities that allow their expression both extra- and intracellularly, their small size favoring tissue penetration, easy engineering allowing the generation of multimeric constructs, low immunogenicity due to their high similarity to the variable heavy domains of human antibodies and versatility in antigen recognition (5–7).

Intrinsic stability is of particular importance for the construction of synthetic Nb libraries (8), as well as for *in vitro* affinity maturation and the engineering of these molecules in general. Several studies have been performed aiming to understand both sequence and structural factors related to Nb stability, especially thermostability as measured through the melting temperature (*T*_m_) (7, 9–14), which is the temperature at which approximately 50% of the protein molecules are unfolded. This property is closely related to other attributes such as solubility and cell production. Thus, various studies have shown that mutation of one or several residues can increase *T*_m_ but drastically decrease yield (15, 16), while in other cases, it can affect the affinity for the antigen (15, 17–20).

The scarce availability of thermostability data for Nbs makes it difficult to generate algorithms to predict their *T*_m_. Currently, several Nb databases have been reported, such as the ‘Integrated Nanobody Database for Immunoinformatics’ (21), SAbDab-nano (22), CoV-AbDab (23) and the ‘Institute Collection and Analysis of Nanobodies’ (iCAN) (24). These databases are focused mainly on sequences and crystallographic structures, although CoV-AbDab and iCAN also contain data on antigens and binding affinities. In this work, we present NbThermo—the first database that collects thermostabilty data for hundreds of Nb, together with amino acid sequences, 3D structures and other data that we considered important for studies that aim to understand the determinants of Nb stability.

## Methods

### Data collection

We started with an exhaustive search of the available literature in PubMed and Google Scholar, using the following keywords: VHH, sdAbs, sybody, nanobody, HCAb, single domain antibody, antibody fragment, thermal stability and melting. With these keywords, we attempted to collect all the publications containing together any of the terms used for Nbs and thermostability data. Our search covered the time interval between 1993 (the year when HCAbs in camelids were first reported) and 10 October 2022.

The following data were compiled for each database entry:

- Name of the Nb.
- Reference DOI.
- Crystallographic structure (Protein Data Bank entry) when available.
- Melting temperature (*T*_m_), subclassified according to the employed experimental method: nano differential scanning fluorimetry (nanoDSF), DSF with SYPRO Orange, differential scanning calorimetry (DSC), circular dichroism (CD) + refolding and ‘Other’ for methods not mentioned earlier.
- Antigen, with the following fields: name, type and affinity.
- Cell production, subclassified according to the cellular location: periplasm, cytoplasm, and other for those without this information.
- Origin, subclassified into source for the organism (generic name, includes synthetic and unknown); type, according to whether the Nb is natural, fully synthetic or semi-synthetic; and obtaining method, which comprises immunization, naive, point mutation or other modification procedures.
- Sequence, with the following fields: raw sequence, sequence numbered using the Aho scheme (25), framework (Fw) and complementarity-determining regions (CDRs), labeled as follows: Fw1, CDR1, Fw2, CDR2, Fw3, CDR3, Fw4.

### Annotations

Data found in text mode (e.g. Nb name, antigen name, DOI, sequence, etc.) were copied manually directly from the sources. Most of the sequences reported in the literature are found as images, and therefore, an online optical character recognition-to-text conversion tool (https://www.ocr2edit.com/convert-to-txt) was used, followed by a careful visual inspection of the obtained text. Each sequence, as well as mutant constructs whose sequences were not explicitly reported, was thoroughly checked. Likewise, antigen data such as affinity values (*K*_D_—dissociation constant) as well as *T*_m_ and expression yields were manually annotated from tables in research articles and supplementary materials. *K*_D_ values were standardized in nanomolar (nM), while the expression yields were standardized in mg/l. The amino acid sequences were numbered according to the Aho scheme (25) using the ANARCI tool (26).

### Implementation of NbThermo

A static website hosted on GitHub (https://github.com/) was created to implement and provide free access to NbThermo (https://valdes-tresanco-ms.github.io/NbThermo). The frontend for this project was generated using Angular CLI version 13.2.4 (https://angular.io/cli).

## Results

### General overview

The literature search yielded ca. 1200 articles, which were all reviewed. As result, we collected data on 564 Nbs, found in 65 publications.

### Melting temperature

**Figure 1.**
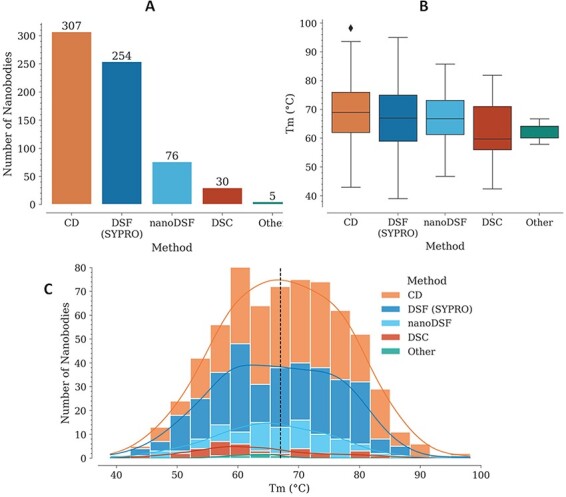
*T*_m_ data in the NbThermo database. (A) Distribution of *T*_m_ measurements by experimental methods: CD, DSF with SYPRO Orange, nanoDSF and DSC. (B) Distribution of *T*_m_ values as measured using different experimental methods. The box represents the interquartile range, the horizontal line within the box represents the median and the bottom and upper lines account for the minimum and maximum *T*_m_, respectively. (C) Distribution of *T*_m_ values across the database, showing with stacked bars the use of the different experimental methods. The median value is highlighted by a vertical dashed line.

For each Nb, *T*_m_ is reported with also the experimental method used for its measurement. In several cases, different values are reported for two or more experimental methods, for the same Nb. The method with the highest representation is CD, followed by DSF with SYPRO Orange, nanoDSF, DSC and a few cases where the experimental method is not reported (Figure 1A).

In most cases where *T*_m_ was measured using CD, the refolding ratio (secondary structure recovery) is also reported, evidencing that Nbs with high melting temperatures regain most of their secondary structure, with only a very few exceptions (data not shown). While *T*_m_ experiments using CD require specialized equipment, which limits its use, DSF with SYPRO Orange is more accessible since it can be performed with real-time PCR (RT-PCR) equipment and can cover a similarly broad *T*_m_ range (Figure 1B). Unlike with CD, Nb denaturalization with the SYPRO Orange reagent is irreversible, since it binds to the hydrophobic core of the structure. This is why using this method, it is not possible to measure the refolding ratio, but nonetheless, due to its higher accessibility, lower costs and ease of execution, its use for *T*_m_ determination is increasing.

Individually, the different experimental methods for *T*_m_ measurement show similar medians across the database: 69, 67, 67, 60 and 64°C, for CD, DSF (SYPRO), nanoDSF, DSC and ‘Other’, respectively (Figure 1B). Considering all the experimental methods, *T*_m_ is in the range from 39 to 98°C, with a median of 67.2°C (Figure 1C).

### Nb origin and sequence

**Figure 2.**
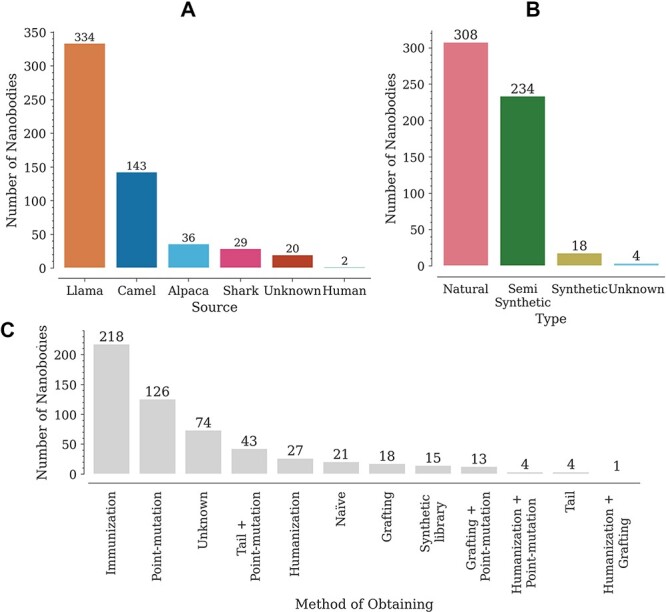
Distribution of Nbs in the database according to (A) source (organism), (B) type and (C) obtaining method.

The database contains a wide variety of Nbs, coming mainly from llamas (302) and camels (175) (Figure 2A). The variable domains of the new antigen receptor of sharks, also included in the database, differ in sequence from camelid Nbs. Most of the reported Nbs are of natural origin, obtained from immune or naïve libraries (Figure 2B). Here, we have regarded as semi-synthetic all Nbs with at least one engineered modification, such as point mutations or tail additions (Figure 2B). It is worth noting that, although an N- or C-terminal addition does not modify the Nb sequence, it does have an effect on *T*_m_. The Nbs in the database were obtained by multiple methods, with the most common being immunization and site-directed mutagenesis (point mutations) (Figure 2C). Animal immunization has been a widely used method to obtain high affinity binders, for which *T*_m_ is reported as stability data. Introducing point mutations in some structures, on the other hand, aims at improving Nb–antigen affinity (*in vitro* affinity maturation) and/or improving other properties such as thermostability, solubility or cell production (7, 10, 12, 15, 16).

Manual annotation and revision of the amino acid sequences were undoubtedly the most complex task in the construction of this database. All the original sequences, including those with various tails such as His, Myc and Hop tags, were annotated. In addition, the Nb sequences were numbered according to the Aho scheme (25), which we selected among others mainly because it distributes the amino acids symmetrically around the gaps in the CDRs, thus piling up in the alignment those amino acids that are structurally close. This is useful especially for phylogenetic analyses of positional amino acid conservation/variation. Other numbering schemes will be added in the future.

**Figure 3.**
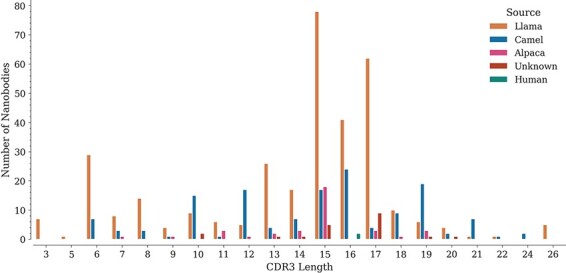
Distribution of CDR3 lengths in the database by source organism.

For each sequence, we also annotated the lengths of its three CDRs. While CDR1 and CDR2 do not show important length variations (data not shown), CDR3 lengths span a wide range, from 3 to 26 amino acids, with lengths 15, 16 and 17 being the most represented in the database (Figure 3), similarly as reported in the recently published ‘Integrated nanobody database for immunoinformatics’ (21).

### Other annotations

NbThermo includes also data related to Nb engineering. While mutations in the Fw region would rather not affect the affinity, mutations generated in the *in vitro* affinity maturation process may affect thermostability. We then included annotations on reported antigens (mostly of protein nature) and their affinities. Furthermore, some mutations can affect cell production, and therefore, we also included these data when available.

### Implementation as website

**Figure 4.**
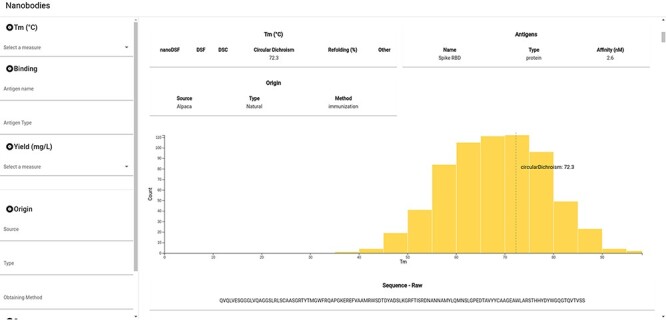
Snapshot of the NbThermo database interface. The left panel contains the annotation filters, while the right panel displays the selection list and the annotated information corresponding to the selected Nbs.

NbThermo is freely available on its website (https://valdes-tresanco-ms.github.io/NbThermo) and in the repository (https://github.com/Valdes-Tresanco-MS/NbThermo). The graphical interface contains two panels (Figure 4): the left panel shows annotation filters (left) and the right panel shows the selection list and the annotated information, where the information corresponding to the selected Nbs is given. The filter panel allows the user to explore on the fly amino acid sequences, *T*_m_ ranges, experimental methods, antigens and any other field in the database.

For each selected Nb, the data registered in the database are shown in the form of cards. In the same way, the *T*_m_ for the selected entry is highlighted in the *T*_m_ distribution graph of all the registered Nbs. Furthermore, users can access the original sequence, with its both sequential and Aho numbering schemes and information on the CDR and Fw regions. Finally, if available in the Protein Data Bank, the Nb three-dimensional structure can be inspected with an embedded molecular visualizer.

### Exploring possible determinants of Nb thermostability

As an exploratory analysis of the data compiled in NbThermo, we set to investigate three questions of scientific and practical relevance, counting now with a richer source of data to support the analyses.

#### Q1—are there differences in the thermostability distribution for Nbs belonging to distinct species?

**Figure 5.**
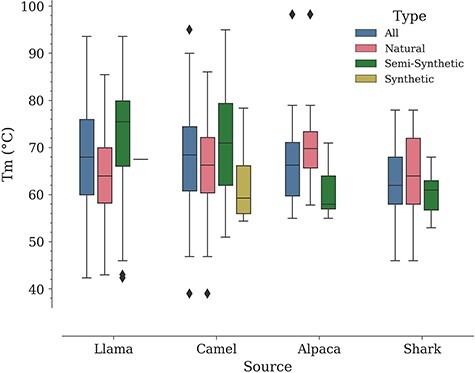
Distribution of *T*_m_ values by Nb: source and type.

In a study by Kunz *et al.*, carried out with a dataset of 78 Nbs (camel—57, llama—17, alpaca—4, all of them of natural origin according to our classification), it was noted that llama Nbs tend to have a higher median *T*_m_ compared to camel Nbs (9). Performing a similar analysis in NbThermo yields the opposite result, although with a minor difference. Natural Nbs from camels show a higher median *T*_m_ (66 vs. 64°C for llama Nbs). However, if we include also Nbs of other origins, the overall medians for camel and llama Nbs are close to each other (camel *T*_m median_ = 68.5°C; llama *T*_m median_ = 68.0°C), followed by the median *T*_m_ values for alpacas and sharks (66 and 62°C, respectively) (Figure 5). Noteworthily, all organisms show a similar distribution around the overall database mean *T*_m_ value (67°C) (Figure S1). Since the difference between the median *T*_m_ of camels and llamas is small (2°C), the selection of one or the other species as a source of Nbs would be rather determined by other factors such as production costs.

#### Q2—is there a correlation between thermostability and CDR3 length?

We hypothesized that Nbs having longer CDR3, which are mostly bent over a Fw surface area that in classical antibodies interacts with the variable light domain, would show higher *T*_m_ values precisely because of the CDR3 packing against the Fw. However, only a poor correlation was obtained, which was slightly higher for camel Nbs (Figure S2). These results are similar to those obtained by Kunz *et al.* (7) using a smaller dataset.

#### Q3—are there different sequence patterns among the frameworks of Nbs with low and high thermostabilities?

**Figure 6.**
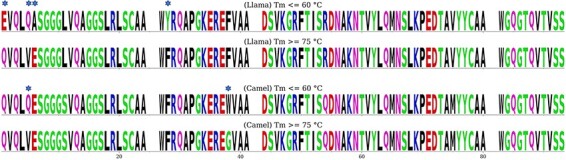
Llama and camel consensus Fw sequences for the low- and high-*T*_m_ groups. The white spaces correspond to the three CDRs. Sequence positions differing between the two groups of the same species are marked with an asterisk.

Identifying sequence patterns in the Fw that would confer high thermostability to Nbs based on such Fws is of outmost importance for Nb engineering, particularly for the design of synthetic libraries. Here, we compared, both for camel and llama Nbs, two groups of Fw sequences having separated ranges of melting temperature: *T*_m_ ≤ 60°C and *T*_m_ ≥ 75°C. For each group, we extracted the consensus Fw sequence (Figure 6).

Interestingly, only four differences in the consensus Fw sequences (E1Q, Q5V, A6E and Y28F) were found between the low- and high-*T*_m_ llama groups, while only two differences were found for the camel groups (Q5V and W38G). It has been shown that even a single mutation may cause a considerable *T*_m_ variation; however, these few differences in consensus sequence can hardly explain the *T*_m_ differences between the low- and high-*T*_m_ groups since the differing amino acids are present in both groups (Figure S3). For example, in camel Nbs, both tryptophan and glycine are found at position 38 in both the low- and high-*T*_m_ groups, although at different frequencies. Interestingly, comparison of the consensus sequences between the high- *T*_m_ groups in llama and camel shows that they differ by the same number of positions as the high- and low-*T*_m_ groups in llama (Table S1).

These findings indicate that the Fw structure alone does not define Nb thermostability, implying that the CDRs make an essential contribution. In particular, the interaction between CDR3 and the Fw may play a significant role, as found in a recent study by Kinoshita *et al.*, showing that mutations of amino acids at this interface can cause a considerable *T*_m_ decrease (27). Thus, the so-called ‘universality’ of a Fw, relevant for synthetic library construction, would then rely on the Fw adaptability to variable CDR3 characteristics, such as length, amino acid composition and conformation.

Noteworthily, when performing a similar sequence comparison for Nbs for which refolding data are available (the analysis was carried out only for llama Nbs since too little data were found for other camelid species), more variability is observed between the consensus Fw sequences of the Nb groups with low (< 50%) and high (> 75%) refolding ratios (Figure S4). Interestingly, most of the observed differences correspond to solvent-accessible positions. A moderate correlation between *T*_m_ and refolding is observed (*r*_P_ = 0.63 and *r*_S_ = 0.64). However, it is difficult to draw meaningful conclusions because of the limited amount of data on folding recovery in the database.

## Projections

The current implementation of NbThermo allows visualization and retrieval of stored data. Various filters can be applied to select sets of Nbs belonging to distinct categories or having melting temperatures within a desired range. Several features will be improved in the future, such as interactive search based on sequence identity, annotation of new entries by community contributions, artificial intelligence–based structural models and *T*_m_ prediction for new sequences.

## Conclusions

Here, we presented NbThermo—a curated thermostability database of Nbs. The database contains up-to-date data for 564 Nbs, which were compiled from diverse sources and manually annotated to ensure maximum reliability. Our exploratory research, now using a much larger amount of data compared to previous studies, indicates that understanding the structural bases of Nb thermostability is a complex task since both the Fw and the CDR regions play significant roles and there are no evident differences in sequence patterns between the Fws of Nbs with lower and higher melting temperatures. We have put effort in compiling these data hoping to contribute to the development of new algorithms for reliable *T*_m_ prediction, as useful tools to assist Nb engineering for the wide range of applications of these unique biomolecules.

## Supplementary Material

baad021_Supp

## Data Availability

The NbThermo database is available at https://valdes-tresanco-ms.github.io/NbThermo.

## References

[1] van Audenhove I. and Gettemans J. (2016) Nanobodies as versatile tools to understand, diagnose, visualize and treat cancer. *EBioMedicine*, 8, 40–48. 27428417 10.1016/j.ebiom.2016.04.028 PMC4919472

[2] Miller T.W. and Messer A. (2005) Intrabody applications in neurological disorders: progress and future prospects. *Mol. Ther.*, 12, 394–401. 15964243 10.1016/j.ymthe.2005.04.003

[3] Lo A.S.Y. , Zhu Q. and Marasco W.A. (2008) Intracellular antibodies (intrabodies) and their therapeutic potential. *Handb Exp. Pharmacol.*, 181, 343–373. 10.1007/978-3-540-73259-4_15 18071953

[4] Steeland S. , Vandenbroucke R.E. and Libert C. (2016) Nanobodies as therapeutics: big opportunities for small antibodies. *Drug Discov. Today*, 21, 1076–1113. 27080147 10.1016/j.drudis.2016.04.003

[5] Dumoulin M. , Conrath K., van Meirhaeghe A. et al. (2002) Single-domain antibody fragments with high conformational stability. *Protein Sci.*, 11, 500–515. 11847273 10.1110/ps.34602 PMC2373476

[6] Muyldermans S. (2013) Nanobodies: natural single-domain antibodies. *Annu. Rev. Biochem.*, 82, 775–797. 23495938 10.1146/annurev-biochem-063011-092449

[7] Kunz P. , Zinner K., Mücke N. et al. (2018) The structural basis of nanobody unfolding reversibility and thermoresistance. *Sci. Rep.*, 8, 1–10. 29784954 10.1038/s41598-018-26338-z PMC5962586

[8] Valdés-Tresanco M.S. , Molina-Zapata A., Pose A.G. et al. (2022) Structural insights into the design of synthetic nanobody libraries. *Molecules*, 27, 2198. 10.3390/molecules27072198 PMC9000494 35408597

[9] Kunz P. , Flock T., Soler N. et al. (2017) Exploiting sequence and stability information for directing nanobody stability engineering. *Biochim. Biophys. Acta Gen. Subj.*, 1861, 2196–2205. 28642127 10.1016/j.bbagen.2017.06.014 PMC5548252

[10] Goldman E.R. , Liu J.L., Zabetakis D. et al. (2017) Enhancing stability of camelid and shark single domain antibodies: an overview. *Front. Immunol.*, 8, 865. 10.3389/fimmu.2017.00865 PMC5524736 28791022

[11] Hussack G. , Hirama T., Ding W. et al. (2011) Engineered single-domain antibodies with high protease resistance and thermal stability. *PLoS One*, 6, 28218. 10.1371/journal.pone.0028218 PMC3227653 22140551

[12] Zabetakis D. , Anderson G.P., Bayya N. et al. (2013) Contributions of the complementarity determining regions to the thermal stability of a single-domain antibody. *PLoS One*, 8, 1–7. 10.1371/journal.pone.0077678 PMC3797041 24143255

[13] Liu H. , Schittny V. and Nash M.A. (2019) Removal of a conserved disulfide bond does not compromise mechanical stability of a VHH antibody complex. *Nano Lett.*, 19, 5524–5529. 31257893 10.1021/acs.nanolett.9b02062 PMC6975629

[14] Zabetakis D. , Olson M.A., Anderson G.P. et al. (2014) Evaluation of disulfide bond position to enhance the thermal stability of a highly stable single domain antibody. *PLoS One*, 9, e115405. 10.1371/journal.pone.0115405 PMC4272287 25526640

[15] Turner K.B. , Zabetakis D., Goldman E.R. et al. (2014) Enhanced stabilization of a stable single domain antibody for SEB toxin by random mutagenesis and stringent selection. *Protein Eng. Des. Sel.*, 27, 89–95. 24488977 10.1093/protein/gzu001

[16] Liu J.L. , Goldman E.R., Zabetakis D. et al. (2015) Enhanced production of a single domain antibody with an engineered stabilizing extra disulfide bond. *Microb. Cell Fact.*, 14, 158. 10.1186/s12934-015-0340-3 PMC4599338 26449768

[17] Soler M.A. , Fortuna S., de Marco A. et al. (2018) Binding affinity prediction of nanobody-protein complexes by scoring of molecular dynamics trajectories. *Phys. Chem. Chem. Phys.*, 20, 3438–3444. 29328338 10.1039/c7cp08116b

[18] Li T. , Cai H., Yao H. et al. (2021) A synthetic nanobody targeting RBD protects hamsters from SARS-CoV-2 infection. *Nat. Commun.*, 12, 1–13. 34330908 10.1038/s41467-021-24905-z PMC8324831

[19] Hacisuleyman A. and Erman B. (2020) ModiBodies: a computational method for modifying nanobodies to improve their antigen binding affinity and specificity. *J. Biol. Phys.*, 46, 189–208. 32418062 10.1007/s10867-020-09548-3 PMC7334311

[20] Liu J.L. , Shriver-Lake L.C., Anderson G.P. et al. (2017) Selection, characterization, and thermal stabilization of llama single domain antibodies towards Ebola virus glycoprotein. *Microb. Cell Fact.*, 16, 223. 10.1186/s12934-017-0837-z PMC5726015 29233140

[21] Deszyński P. , Młokosiewicz J., Volanakis A. et al. (2022) INDI—integrated nanobody database for immunoinformatics. *Nucleic Acids Res.*, 50, D1273–D1281. 34747487 10.1093/nar/gkab1021 PMC8728276

[22] Dunbar J. , Krawczyk K., Leem J. et al. (2014) SAbDab: the structural antibody database. *Nucleic Acids Res.*, 42, D1140–D1146. 24214988 10.1093/nar/gkt1043 PMC3965125

[23] Raybould M.I.J. , Kovaltsuk A., Marks C. et al. (2021) CoV-AbDab: the coronavirus antibody database. *Bioinformatics*, 37, 734–735. 32805021 10.1093/bioinformatics/btaa739 PMC7558925

[24] Zuo J. , Li J., Zhang R. et al. (2017) Institute collection and analysis of nanobodies (iCAN): a comprehensive database and analysis platform for nanobodies. *BMC Genomics*, 18, 1–5. 29041922 10.1186/s12864-017-4204-6 PMC5646159

[25] Honegger A. and Plückthun A. (2001) Yet another numbering scheme for immunoglobulin variable domains: an automatic modeling and analysis tool. *J. Mol. Biol.*, 309, 657–670. 11397087 10.1006/jmbi.2001.4662

[26] Dunbar J. and Deane C.M. (2016) ANARCI: antigen receptor numbering and receptor classification. *Bioinformatics*, 32, 298–300. 26424857 10.1093/bioinformatics/btv552 PMC4708101

[27] Kinoshita S. , Nakakido M., Mori C. et al. (2022) Molecular basis for thermal stability and affinity in a VHH: contribution of the framework region and its influence in the conformation of the CDR3. *Protein Sci.*, 31, e4450. 10.1002/pro.4450 PMC9601775 36153698

