# Supplementary material for: NbThermo: a new thermostability database for nanobodies
Source: Database (Oxford). 2023 Apr 12;2023:baad021. doi: 10.1093/database/baad021 (PMC10091358; doi:10.1093/database/baad021)
Supplement: baad021_Supp [file baad021_supp.zip › suppl_data/NbThermo_SI_revised.docx]

**NbThermo: a new thermostability database for nanobodies**

Mario S. Valdés-Tresanco^1^*, Mario E. Valdés-Tresanco^2^,

Esteban Molina-Abad^3^, Ernesto Moreno^1^*

1. Faculty of Basic Sciences, University of Medellin, Medellin, Colombia.

2. Centre for Molecular Simulations and Department of Biological Sciences, University of Calgary, Alberta T2N 1N4, Canada.

3. Globant S.A., Medellin, Colombia

Corresponding authors: MSVT, [mariosergiovaldes145@gmail.com](mailto:mariosergiovaldes145@gmail.com); EM, [emoreno@udemedellin.edu.co](mailto:emoreno@udemedellin.edu.co)

**Supplemental information**


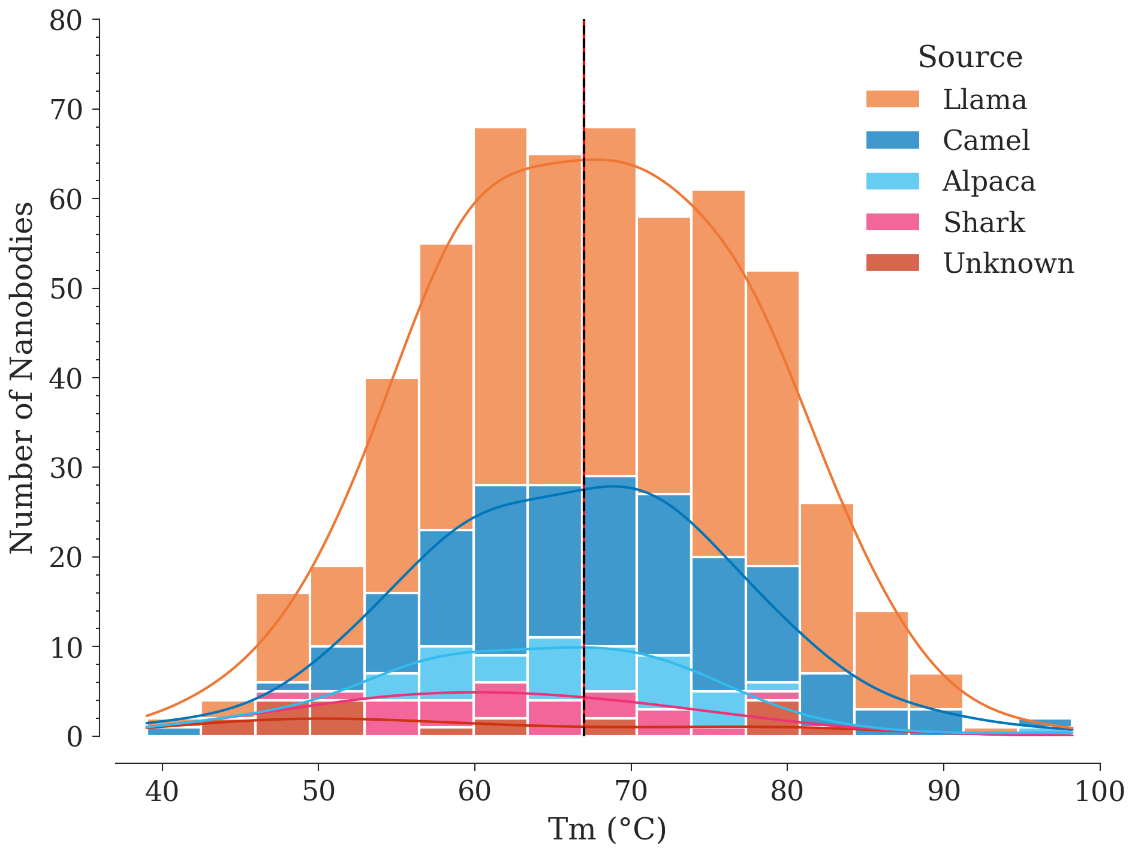


Figure S1. Distribution of Tm values according to the nanobody source. The human VHs were excluded.


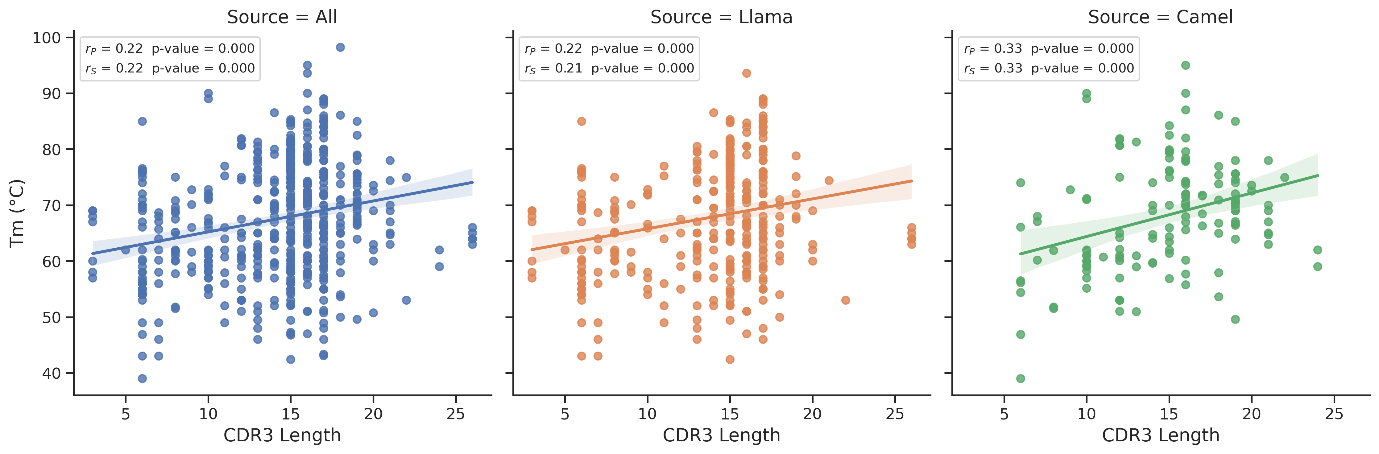


Figure S2. Relationship between CDR3 length and Tm. The first panel from left to right shows the correlation between all the NbThermo Tm values and the CDR3 length, while the second and third panels only include the Llama and Camel data respectively.


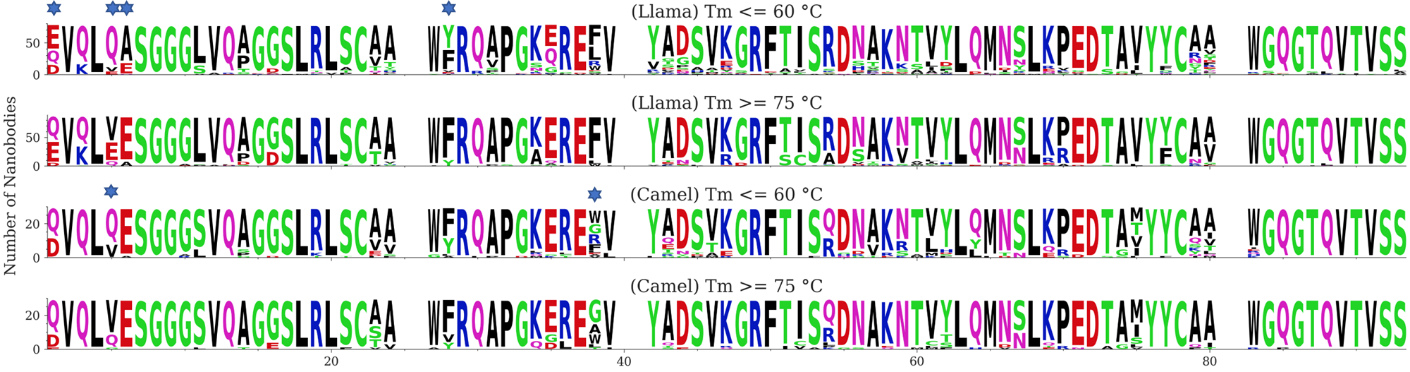


Figure S3. Logos diagram of the framework sequences of the Llama (first and second panel) and Camel (third and fourth panel) nanobodies at low (Tm <= 60 °C) and high (Tm >= 75 °C) Tm. Sequence positions differing between the two groups of the same species are marked with an asterisk


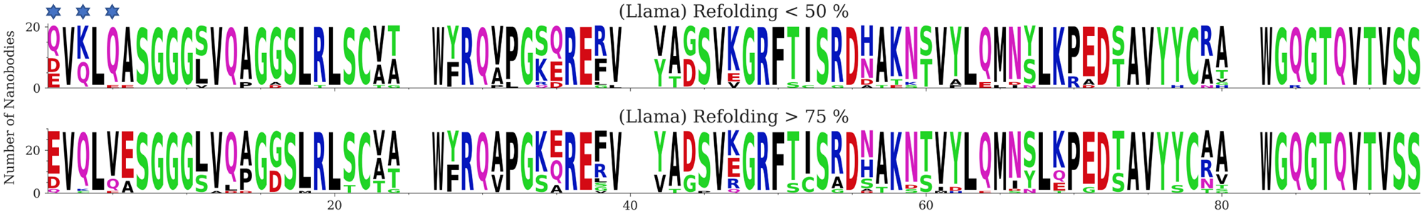


Figure S4. Logos diagram of the framework sequences of Llama nanobodies with low (Refolding < 50%) and high (Refolding > 75%) refolding ratios. Sequence positions that differ between the two groups of the same species and that might play a role in refolding are marked with an asterisk.

Table S1. Number of differences between Llama and Camel consensus framework sequences for low (Tm <= 60 °C) and high (Tm >= 75 °C) Tm.

|  |  | **Llama** | | **Camel** | |
| --- | --- | --- | --- | --- | --- |
|  | Tm | Low | High | Low | High |
| **Llama** | Low |  | 4 | 7 | 8 |
|  | High | 4 |  | 5 | 4 |
| **Camel** | Low | 7 | 5 |  | 2 |
|  | High | 8 | 4 | 2 |  |
